# Oral health status of 12-year-old children in Lisu minority ethnic group in China: a cross-sectional study

**DOI:** 10.1186/s12903-020-01358-2

**Published:** 2021-01-12

**Authors:** Kitty Jieyi Chen, Juan Liu, Biao Xu, Yuexiao Li, Yanhong Li, Shinan Zhang, Chun Hung Chu

**Affiliations:** 1 Department of Pediatric Dentistry, Guanghua School of Stomatology, Hospital of Stomatology, Sun Yat-Sen University, Guangzhou, Guangdong China; 2 Affiliated Stomatological Hospital of Kunming Medical University, Kunming, Yunnan China; 3 Faculty of Dentistry, The University of Hong Kong, Pok Fu Lam, Hong Kong SAR China

**Keywords:** Child, Dental caries, Ethnic group, Minority group, Oral health

## Abstract

**Background:**

Lisu is an ethnic minority group and most of them are living in Yunnan, China. This study investigated the oral health status among 12-year-old Lisu children in Yunnan.

**Method:**

This survey employed a multistage sampling method to recruit 12-year-old Lisu children. Two calibrated dentists performed the oral examinations in the primary schools. They examined dental caries, gingival bleeding and dental fluorosis using the diagnosis criteria recommended by the World Health Organization. A self-administrated questionnaire was distributed to the children to collect their sociodemographic background information and oral health-related behaviours. A chi-square test, the Mann–Whitney U test, zero-inflated negative binomial (ZINB) regression and multivariate logistic regression were used for statistical analysis.

**Results:**

This survey invited 512 children, and 482 children (48% boys) participated in the study (response rate: 94%). Their caries prevalence was 35% and their caries experience in mean (SD) DMFT scores was 0.63 (0.10). The mean (SD) DT score was 0.60 (1.10), consisting 95% of the mean DMFT scores. No dental fluorosis was observed; whereas 426 children (88%) had gingival bleeding. Results of ZINB model indicated sex and sugary-snacking habits were related to the dental caries experience (p < 0.05). The gingival-bleeding prevalence was associated with the mother’s education level, the child’s monthly-pocket money and daily toothbrushing frequency (p < 0.05).

**Conclusion:**

Dental caries and gingival bleeding were prevalent among 12-year-old Lisu children in the Yunnan province in China, and most of the decayed teeth were unrestored. Dental fluorosis was not observed in the children.

## Background

China is a vast country with 1.4 billion people, which consists of 56 ethnic groups [1]. The dominant ethnic group is Han which composes 92% of the population, as reported by the latest census [2]. The other 55 non-Han groups are regarded as ethnic minorities with a total population of more than 110 million people [3]. Ethnic minorities mainly reside in the west and the northeast part of China, particularly in underdeveloped and mountainous areas [4]. Yunnan is a southwest province of China that borders Vietnam, Laos and Myanmar on the west. There are 25 ethnic minorities residing in Yunnan, one of which is the Lisu ethnic minority [5].

The Lisu people are a Tibeto-Burman ethnic group living in Myanmar, southwest China, Thailand and the Indian state of Arunachal Pradesh. They have their own Lisu language and culture. It is estimated that there are 1.5 million Lisu people around the world. Nearly half of them (0.7 million) live in China, ranking as the 20th largest ethnic minority group in that country [6]. According to the fifth census, 96% of the Lisu ethnic minority in China lived in Yunnan, mainly in the mountainous areas along the border of Myanmar [7]. This population remained underdeveloped. In 2018, the disposable income per capita of Lisu people was reported to be 6476 Chinese yuen (~ US$ 913) [8], an amount much lower than that of the national average (~ US$ 4165) [9].

In recent years, the Chinese government has actively invested in Lisu’s infrastructure, agriculture, education and health services to support the development of this underdeveloped population. Oral health is a key indicator of people’s general health, their well-being and quality of life. Poor oral health not only causes pain but also increases the financial burden for society [10]. To improve Lisu people’s oral health status, oral health service is an essential part of the health services. Before planning and implementing a beneficial oral health services scheme for Lisu people, it is necessary to understand their current oral health status. Recommended by World Health Organization (WHO), 12-year-old children are the index age group for monitoring oral health status [11]. At the age of 12, all permanent teeth except the third molar have been erupted. Thus, the oral health status information of 12-year-old Lisu children can be used not only to compare with the oral health situation of the same age group in other countries, but also to help dental researchers and local government design dental services for school children.

China has been conducting national oral health surveys every 10 years and the latest survey was conducted in 2015. The results indicated that more than one fourth of the 12-year-old Chinese children had dental caries experience, more than a half of them had gingival bleeding and 13% of the children were diagnosed as dental fluorosis [12–14]. However, the national survey was conducted based on provinces (geographical administration region) instead of ethnicity. There is no data about the 12-year-old Lisu children’s oral health status until now. The present study aims to investigate the oral health status among the 12-year-old Lisu children in Yunnan. The status of dental caries, gingival bleeding and dental fluorosis were assessed. The secondary outcome of this study was the status of dental erosion and tetracycline stained teeth.

## Method

This cross-sectional study was conducted in 2016 with the support from the Education Bureau of Yunnan Province. Ethical approval was obtained from the Kunming Medical University Institutional Review Board. Invitation letters were sent to legal guardians and written consents were collected. The report of this study follows the Statement of Strengthening the Reporting of Observational Studies in Epidemiology (STROBE) [15].

### Sample size calculation and sample selection

The sample size calculation was based on formula: n = Z^2^ × P × (1 − P)/d^2^, in which P was the prevalence rate of oral diseases, the Z statistic was based on the confidence level and d was the precision [16]. The prevalence rate of oral diseases (P) was estimated to be 50% to yield the maximum sample size. With the confidence level of 95% and the confidence interval of 5% (confidence interval: 45–55%), sample size was calculated to be 384. Estimating a response rate of 80%, at least 480 children needed to be invited in the study.

This study adopted a multistage cluster sampling method. Yunnan has eight cities and eight autonomous prefectures. Most of the Lisu population resides in the western region, which includes three cities (Baoshan City, Lijiang City and Lincang City) and five autonomous prefectures (Chuxiong Yi Autonomous Prefecture, Dali Bai Autonomous Prefecture, Dehong Dai and Jingpo Autonomous Prefecture, Diqing Tibetan Autonomous Prefecture and Nujiang Lisu Autonomous Prefecture). Only a few Lisu are scattered in the eastern region, which consists of five cities and three autonomous prefectures. The distribution ratio of the Lisu population in the western region and eastern region was 8:1 [17]. Accordingly, the ratio of children invited from these two regions was determined. At least 427 children should be invited from the western region and 53 should be invited from the eastern region. Lists of primary schools in western and eastern regions were obtained and the schools were numbered sequentially. Then, schools were selected by a simple random sampling method with a list of random numbers generated by a computer. All 12-year-old Lisu children in the selected schools were invited until the number of invitations in that region was fulfilled. The inclusion criteria were 12-year-old Lisu children who were generally healthy without special care needs, prolonged use of medications and severe chronic diseases and had parental consents. Children who were on long-term medications were excluded.

### Questionnaire survey

Self-administrated questionnaires were distributed to the study children and collected with parental consents one day before the oral examination. A research assistant collected the parental consent together with the questionnaire and checked the responses in school. If the research assistant found any missing or inappropriate answers, the participating children were required to filled in or confirmed their answers on the spot. If the study children failed to answer their parents’ education levels, the research assistant would confirm the data with their parents by phone. The questionnaire was adapted from the one used in a previous epidemiological study [17]. It consisted of two parts as follows:

1. Child’s sociodemographic background information: sex, parental education levels and child’s monthly-pocket money; and
2. Child’s oral health-related behaviours: toothbrushing frequency (daily), sugary snacking habit, sour food snacking habit and dental attendance experience.

### Oral examination

Two calibrated dentists (Y.L and S.Z) conducted the oral examination in the primary schools during school hours with the aid of Community Periodontal Index (CPI) probes, dental mirrors and light-emitting diode headlights for intra-oral illumination. S.Z is an experienced dental epidemiologist. Y.L was calibrated with S.Z in the same setting before the study commenced. To assess the color of tooth crown, the children were required to remove food debris and dental plaque before the oral examination. No special toothbrushing technique was introduced. The research assistant reminded the children to keep their mouth clean on the examination day by following their daily oral hygiene practice routine.

The diagnosis criteria of dental caries followed WHO recommendations [11]. Dental caries experience was measured by the decayed, missing and filled teeth (DMFT) index. If a tooth had an unmistakable cavity, undermined enamel or detectable softened floor or wall, it was recorded as a decayed tooth (DT). If a tooth was extracted due to dental caries, it was recorded as a missing tooth (MT). If a tooth was permanently filled without dental caries, it was recorded as a filled tooth (FT). Children’s periodontal status were assessed by the presence of gingival bleeding followed the WHO recommendation and diagnosis criteria. Periodontal pockets were not assessed as the study children were younger than 15 years old [11]. Examiners inserted the tip of CPI probe into the gingival sulcus to explore the full extent of the sulcus with the sensing force of no more than 20 g. The tip of CPI probe was first placed as close as possible to the distal contact point with the probe paralleled to the long axis of the tooth. Then the probe was move gently with short upward and downward movements along the sulcus to the mesial contact point. Gingivae of all teeth present in mouth were examined. Children who had bleeding on probing in any tooth site was diagnosed as having gingival bleeding [11]. Dean’s index criteria were used to assess dental fluorosis as recommended by WHO [18]. Enamel surface that had white flecks, occasional spots, paper-white areas or brown staining was regarded as dental fluorosis. Basic Erosive Wear Examination (BEWE) criteria were adopted in this study to assess the status of dental erosion [19]. All tooth surfaces in six sextants (17–14, 13–23, 24–27, 37–34, 33–43 and 44–47) were screened and the most severely affected tooth surface in each sextant were scored. The scoring consists of four levels: (0) no loss of the tooth surface, (1) initial loss of enamel texture, (2) distinct defect with hard tissue less than 50% of the tooth surface area, and (3) hard tissue loss of more than 50% of the tooth surface area. The cumulative scores of all six sextants were calculated to assess the patient’s risk level. There were four risk levels including none (BEWE score ≤ 2), low (BEWE score = 3–8), medium (BEWE score = 9–13) and high (BEWE score ≥ 14). Tetracycline-stained teeth were diagnosed by assessing the tetracycline discoloration of the tooth’s crown in the daylight [20]. A 10% random sample of the study children were selected by a dental assistant for duplicate examination without notifying the examiners on the same day. The duplicate examination was performed to assess the inter- and intra-examiner agreements.

### Data analysis

Data were analyzed by IBM SPSS version 25.0 (IBM Corp., NY, USA) and SAS® OnDemand for Academics (SAS Inst., NC, USA). The intra- and inter-examiner reliability was assessed by intraclass correlation coefficient (ICC). A chi-square test was conducted to test the association between independent variables and the prevalence of oral conditions (dental caries and gingival bleeding). The Mann–Whitney U test was performed to analyze the distribution of DMFT scores according to different independent variables because the distribution of DMFT score was not normal. This study considered the zero-inflated negative binomial regression model (ZINB) to study the relationships between the DMFT scores and covariates. The Vuong’s test was employed to test the model fit. A multivariate logistic regression model was considered to study the relationship between the prevalence of gingival bleeding and covariates. Independent variables with p-value less than 0.1 in the univariate analysis (chi-square test, Mann–Whitney U test) were studied as covariates in the regression models. Doing so can minimize the influence of the irrelevant variables and prevent missing important variables [21]. Insignificant variables were removed from the models by backward stepwise selection until all remaining variables had a p-value less than 0.05.

## Result

This study invited 512 12-year-old Lisu children from nine primary schools to participate, and 482 children (48% boys) were successfully recruited. All invited schools agreed to participate. Thirty invited children were absent from their respective schools and did not return the parental consent forms. Thus, the response rate was 94%. Among the participating schools, seven were from the western region and two from the eastern region. The ratio of recruited children in the western region and the eastern region was 8:1 (430:52). The intra- and inter-examiners reliability were excellent with the ICC above 0.90 for the assessment of dental caries, gingival bleeding, tetracycline-stained teeth and dental fluorosis. The children’s background information and their oral health-related behaviours are presented in Table 1.

**Table 1:** Socio-demographic background and oral health-related behaviours of the study Lisu children

Variables (N = 482)	n	%
*Socio-demographic background*		
Sex		
Boy	233	48.3
Girl	249	51.7
Father’s education level		
Primary school level, ≤ 6 years	271	56.2
Secondary school level or above, > 6 years	211	43.8
Mother’s education level		
Primary school level, ≤ 6 years	282	58.5
Secondary school level or above, > 6 years	200	41.5
Monthly pocket money		
Less than 50RMB (~ US$ 7)	325	67.4
More than 50RMB (~ US$ 7)	157	32.6
*Oral health-related behaviours*		
Toothbrushing frequency (daily)		
Less than twice	315	65.4
Twice or above	167	34.6
Sugary snacking habits		
No	335	69.5
Yes	147	30.5
Sour food snacking habits		
No	360	74.7
Yes	122	25.3

*RMB* Renminbi (Chinese Yuen), *US$* U.S. Dollar

### Oral health status

Among the study children, 167 (34.6%) had dental caries experience. The girls had a higher dental caries prevalence rate than boys (42% vs. 27%, p < 0.001, Table 2). The mean (standard deviation [SD]) DMFT score was calculated to be 0.63 (1.10). The majority (95.2%) of the mean DMFT score consisted of unrestored cavities with the mean DT score of 0.60 (SD: 1.10). The mean (SD) MT score was 0.02 (0.13), and the mean (SD) FT score was 0.01 (0.16). First molars had the highest dental caries prevalence rate (58.8%), while canines were the least affected ones (0.4%). The prevalence rate for all incisors and premolars were lower than 10%.

**Table 2:** Prevalence of dental caries and gingival bleeding according to socio-demographic background and oral-health related behaviours

Variables (N = 482)	DMFT > 0 (%)	p-value	Gingival bleeding (%)	p-value
*Socio-demographic background*				
Sex		< 0.001*		0.791
Boy (233)	27		88	
Girl (249)	42		89	
Father’s education level		0.345		0.003*
Primary school level, ≤ 6 years (271)	33		92	
Secondary school level or above, > 6 years (211)	37		83	
Mother’s education level		0.361		< 0.001*
Primary school level, ≤ 6 years (282)	33		93	
Secondary school level or above, > 6 years (200)	37		82	
Monthly pocket money		0.120		< 0.001*
Less than 50RMB (~ US$ 7) (325)	32		94	
More than 50RMB (~ US$ 7) (157)	39		77	
*Oral health-related behaviours*				
Toothbrushing frequency (daily)		0.667		< 0.001*
Less than twice (315)	34		93	
Twice or above (167)	36		80	
Sugary snacking habits		0.142		0.129
No (335)	33		90	
Yes (147)	40		85	
Sour food snacking habits		0.298		0.057
No (360)	33		90	
Yes (122)	39		84	

*Statistically significant difference between groups

*DMFT* decayed, missing and filled teeth, *RMB* Renminbi (Chinese Yuen), *US$* U.S. Dollar

For gingival bleeding, 426 children (88.4%) had gingival bleeding by probing. No significant difference was found between boys and girls regarding the prevalence of gingival bleeding (Table 2). First molars presented the highest prevalence rate of gingival bleeding (65.3%) and canines had the lowest prevalence rate (24.3%). However, unlike that of dental caries, all four lower incisors presented high prevalence rates, meaning over 30%. This study found no relationship between gingival bleeding and dental caries (p = 0.438). None of the study children had dental fluorosis.

There were 53 children who were not cooperative during the dental erosion status assessment. Therefore, dental erosion was assessed for 429 children. All study children had dental erosion, but none of them had severe erosion (BEWE score = 3). Twenty-three children (5.4%) had at least one sextant that had distinct defect of the tooth surface (BEWE score = 2), and 428 children (99.8%) had at least one sextant with an initial loss of enamel surface texture (BEWE score = 1). The cumulative scores of all sextants ranged from 2 to 12. Only one child had no risk with the cumulative BEWE score equaled to two. Almost all children (n = 425, 99.1%) had low risk with the cumulative BEWE score between three and eight, while three were at medium risk with the score between 9 and 12. Almost all of the study children did not have tetracycline-stained teeth (99.3%). The three children (0.7%) who had tetracycline-stained teeth were all from the western region but in different cities/autonomous prefectures.

### Risk factors for dental caries and gingival bleeding

In the chi-square test of independent variables and the prevalence of dental caries, only sex had a p-value less than 0.10 (Table 2). In the Mann–Whitney U test of independent variables and the mean rank of the median DMFT scores, sex and sugary snacking habits were found to have a p-value less than 0.10. These mentioned independent variables were studied as covariates in the ZINB model. The results of the Voung test showed that the ZINB model is the best-fit model when compared to other count models (p < 0.05). In the final model, girls were found to have less chance to have no dental caries when compared to boys (Odds ratio [OR] = 0.35, p = 0.012). In the negative binomial portion of the model, children who had a sugary snacking habit presented higher DMFT scores compared to those who did not (Incidence risk ratio [IRR] = 1.55, p = 0.005) (Table 3).

**Table 3:** Dental caries risk factors of the 12-year-old Lisu children (zero inflated negative binomial regression model, N = 482)

Risk factor	Odds ratio (95% C.I)	p-value
Zero-inflated portion (DMFT = 0)		
Sex		
Girl	0.35 (0.15, 0.79)	0.012
Boy^a^		

Risk factor	Incidence rate ratio (95% C.I)	p-value
Negative binomial portion (DMFT > 0)		
Sugary snacking habits		0.005
Yes	1.55 (1.14, 2.11)	
No^a^		

*DMFT* decayed, missing and filled teeth

^a^Reference group

For gingival bleeding, five independent variables had a p-value less than 0.10 in the chi-square test of independent variables and gingival bleeding (Table 2). They were studied as covariates in the multivariate logistic regression model. In the final model, three variables were significantly related to the prevalence of gingival bleeding (Table 4). Children whose mothers had a lower education level and those who had less pocket money had a higher chance of having gingival bleeding (OR = 2.51, p = 0.003 and OR = 3.75, p < 0.001, respectively). Moreover, children who brushed their teeth less than twice daily had a higher chance of having gingival bleeding (OR = 2.41, p = 0.004).

**Table 4:** Gingival bleeding risk factors of the 12-year-old Lisu children (multivariate logistic regression model, N = 482)

Risk factors	Odds ratio (95% C.I.)	p-value
Mother’s education level		
Primary school level, ≤ 6 years	2.51 (1.32, 3.53)	0.003
Secondary school level or above, > 6 years^a^		
Monthly pocket money		< 0.001
Less than 50RMB (~ US$ 7)	3.75 (2.05, 6.86)	
More than 50RMB (~ US$ 7) ^a^		
Toothbrushing frequency (daily)		0.004
Less than twice	2.41 (1.33, 4.39)	
Twice or above^a^		

*RMB* Renminbi (Chinese Yuen), *US$* U.S. Dollar

^a^Reference group

## Discussion

This study is the first epidemiological study to investigate the oral health status among 12-year-old Lisu children in China. To maintain the representativeness, this study employed a multistage sampling method to recruit children in primary schools as home schooling is not common in China. This sampling technique is convenient and cost-effective in terms of recruitment, especially for the Lisu population who resides in remote and mountainous villages. However, this sampling technique may not be as precise as a simple random sampling [11]. Despite this, this study successfully recruited children from primary schools in different cities/autonomous prefectures and achieved a high response rate. To yield an optimal sample size, this study set P (prevalence) equal to 50% because it was impossible to come up with a good estimate for P from previous study [16]. The sample size in this study was large enough for dental caries and gingival bleeding. However, a larger sample size or a census may be conducted to study the rare oral diseases among the study group. To maintain the reliability and validity, two examiners underwent sufficient calibration training in the same setting before the study commenced, and they obtained very good inter- and intra- examiner agreements. They also adopted the diagnosis criteria suggested by WHO, and the results of this study can be used to compared with other epidemiological studies. In addition, this study assigned a research assistant to check the questionnaires and followed up the missing and inappropriate answers on the spot to reduce non-response and response bias. This study found that dental caries, gingival bleeding and dental erosion were prevalent among 12-year-old Lisu children, while tetracycline-stained teeth and dental fluorosis were not prevalent.

When compared to the national data of 12-year-old children in China, Lisu children presented higher prevalence rates in dental caries and gingival bleeding. The dental caries prevalence rate of Lisu children was 35%, which is slightly higher than that of the national average (24%) [12]. Along with this, over 95% of the cavities were unrestored. For gingival bleeding, the prevalence rate of Lisu children was much higher than the national data (88% vs. 58%) [13]. The high prevalence rate of dental caries and gingival bleeding suggested that inequalities in oral health might exist among the Lisu children. Generally, these Lisu children were from the low socio-economic class in terms of family disposable income per capita and area-level socio-economic status (rural villages) [8, 9]. Besides, dental treatment or clinical prevention treatment may be neither available nor affordable for the Lisu children in the mountainous area. The barrier to dental care also increased the inequality in oral health [22]. Around half of the children in this study had never visited a dentist. Therefore, there is an urgent need to reduce the oral health inequalities among the Lisu ethnic minority for the purpose of fairness and social justice. In 2016, The General Office of the State Council of China issued the Healthy China Plan for 2030, which provides oral health education, oral hygiene instruction and pit and fissure sealant to all 7- to 9-year-old children in China [23]. The effectiveness of this oral health promotion programme on Lisu children’s oral health needs to be confirmed.

This study adopted the ZINB model to analyze the relationship between covariates and DMFT scores. The zero-inflated part of ZINB model indicated the relationship between covariates and the presence of caries (have dental caries or not). The negative binomial part of ZINB model studied the association between positive caries experience (dmft > 0) and covariates [21]. Independent variables with p-value less than 0.10 in chi-square test fitted in the zero-inflated part and those with p-value less than 0.10 in Mann–Whitney U test fitted in the negative binomial part. Two variables were found to be related to the prevalence of dental caries, including sex and sugary snacking habits. Females were typically found to have a higher prevalence rate due to their hormonal fluctuations [24]. For sugary snacks habits, these study Lisu children live in the subtropical zone where sugar is produced, and the readily available sweet foods or drinks in their daily life was likely to increase the risk of dental caries [25]. All these risk factors should be taken into consideration for the Lisu population’s best benefit. Special dental care for female and sugar control should be supported by the local government. For gingival bleeding, the frequency of toothbrushing was related to its prevalence. Gingival bleeding is an indicator of periodontal disease which is reversible by maintaining good oral hygiene, and toothbrushing is the most common oral-hygiene practice [26]. However, more than half of the study children did not have regular twice-daily toothbrushing habits. Therefore, a school-based oral healthcare instruction and regular reinforcement in toothbrushing should be considered to improve the situation [27]. But the oral hygiene status (the presence of dental plaque) of the study children was unable to assess, which was the limitation of this study. The reason was all children were required to brush their teeth before the oral examination to assess the color of tooth crown for tetracycline-stained teeth and dental fluorosis. Along with this, the relationship between dental plaque and dental caries or gingival bleeding among the study children was unable to be analysed.

Another issue to be aware of is that all of the study Lisu children had dental erosion, though none of them were diagnosed as severe dental erosion and just a few were moderate dental erosion. Further studies should be conducted to investigate the reason for this epidemic oral condition and prevention strategies should be developed. Some children did not finish the dental erosion assessment due to the length of oral procedure, which might have influenced the results. But the representativeness should still be maintained with a large sample size. In addition, some Lisu children did have tetracycline-stained teeth even though tetracycline was not used commonly in recent years due to the side effects [20]. Restrictions on prescriptions of this kind of antibiotics for young children should be imposed. For dental fluorosis, cautions should be taken when interpreting the results. Sampling in this study collected information from a part of the whole Lisu population and it might be unable to gather information for every member of the population. Therefore, dental fluorosis might still present on a small number of children. Nevertheless, this study still adds information to the literature, and stakeholders can have a better understanding of the oral health status of 12-year-old Lisu children.

## Conclusion

Dental caries and gingival bleeding were prevalent among 12-year-old Lisu children in the Yunnan province of China, and most of the decayed teeth were unrestored. Dental fluorosis was not observed in the children.

## Data Availability

The datasets used for the current study are available from the first author on reasonable request.

## Abbreviations

BEWE: Basic Erosive Wear Examination
CPI: Community Periodontal Index
DMFT: Decayed, missing and filled teeth
DT: Decayed tooth
FT: Filled tooth
MT: Missing tooth
RMB: Renminbi (Chinese Yuen)
SD: Standard deviation
STROBE: Statement of Strengthening the Reporting of Observational Studies in Epidemiology
US$: United States dollar
WHO: World Health Organization
ZINB: Zero-inflated negative binomial regression

## References

[1] Countrymeters. China Population. 2020. https://countrymeters.info/en/China. Accessed 30 Apr 2020.

[2] National Bureau of Statistics. The main data report of the sixth census in 2010 [In Chinese]. 2011. http://www.stats.gov.cn/tjsj/tjgb/rkpcgb/qgrkpcgb/201104/t20110428_30327.html. Accessed 30 Apr 2020.

[3] Mackerras C, Zang X (2015). Ethnic minorities. Understanding Chinese Society.

[4] Zheng C (2013). Population increase and distribution of ethnic minorities in China since 2000 [in Chinese]. Northw Popul J.

[5] The Central People’s Government of the People’s Republic of China. Yunnan [In Chinese]. 2013. http://www.gov.cn/test/2013-03/28/content_2364754.htm. Accessed 12 May 2020.

[6] The Central People’s Government of the People’s Republic of China. Lisu ethnic group [In Chinese]. 2020. http://www.gov.cn/guoqing/2015-07/24/content_2902168.htm. Accessed 17 Apr 2020.

[7] Hao S, Ren Y, Chen Y (2002). Atlas of distribution of national minorities in China.

[8] Xinhuanet. Fugong county in Yunnan: Lisu people’s new life in Ganbu Village [in Chinese]. 2019. http://www.xinhuanet.com/politics/2019-11/03/c_1125186229.htm. Accessed 28 Apr 2020.

[9] CEIC. China Business Sales of Key Circulation Enterprise. 2019. https://www.ceicdata.com/en/china/business-sales-of-key-circulation-enterprise. Accessed 28 Apr 2020.

[10] World Health Organization. Oral Health. 2020. https://www.who.int/health-topics/oral-health/#tab=tab_1. Accessed 1 Sep 2020.

[11] World Health Organization (2013). Oral health surveys: basic methods.

[12] Quan JK, Wang XZ, Sun XY, Yuan C, Liu X, Wang X (2018). Permanent teeth caries status of 12-to 15-year-olds in China: findings from the 4th National Oral Health Survey. Chin J Dent Res.

[13] Chen X, Ye W, Zhan JY, Wang X, Tai BJ, Hu DY (2018). Periodontal status of Chinese adolescents: findings from the 4th National Oral Health Survey. Chin J Dent Res.

[14] Zhou Y, Chen DR, Zhi QH, Tao Y, Wang X, Feng XP (2018). The prevalence and associated risk indicators of dental fluorosis in China: findings from the 4th National Oral Health Survey. Chin J Dent Res.

[15] Von Elm E, Altman DG, Egger M, Pocock SJ, Gøtzsche PC, Vandenbroucke JP (2014). The Strengthening the Reporting of Observational Studies in Epidemiology (STROBE) Statement: guidelines for reporting observational studies. Int J Surg.

[16] Naing L, Winn T, Rusli BN (2006). Practical issues in calculating the sample size for prevalence studies. Arch Orofac Sci.

[17] Zhang S, Li Y, Liu J, Wang W, Ito L, Li SKY (2019). Dental caries status of Lisu preschool children in Yunnan Province, China: a cross-sectional study. BMC Oral Health.

[18] Dean HT, Moulton F (1942). Fluoride and dental health. The investigation of physiological effects by the epidemiological method.

[19] Bartlett D, Ganss C, Lussi A (2008). Basic Erosive Wear Examination (BEWE): a new scoring system for scientific and clinical needs. Clin Oral Investig.

[20] Sánchez AR, Rogers RS, Sheridan PJ (2004). Tetracycline and other tetracycline-derivative staining of the teeth and oral cavity. Int J Dermatol.

[21] Bursac Z, Gauss CH, Williams DK, Hosmer DW (2008). Purposeful selection of variables in logistic regression. Source Code Biol Med.

[22] Sheiham A, Conway D, Chestnutt I, Watt RG, Listl S, Peres M, Heilmann A (2015). Social inequalities in oral health: from evidence to action.

[23] The Central People’s Government of the People’s Republic of China. Healthy China Campaign (2019–2023) [in Chinese]. http://www.gov.cn/xinwen/2019-07/15/content_5409694.htm. Accessed 30 Apr 2020.

[24] Lukacs JR, Largaespada LL (2006). Explaining sex differences in dental caries prevalence: saliva, hormones, and “life-history” etiologies. Am J Hum Biol.

[25] World Health Organization (2017). Sugars and dental caries.

[26] Kinane DF, Attström R (2005). European Workshop in Periodontology group B: advances in the pathogenesis of periodontitiss: group B consensus report of the fifth European workshop in periodontology. J Clin Periodontol.

[27] Livny A, Vered Y, Slouk L, Sgan-Cohen HD (2008). Oral health promotion for schoolchildren—evaluation of a pragmatic approach with emphasis on improving brushing skills. BMC Oral Health.

